# Pancreatic Adenosquamous Carcinoma Discovered Upon a Resection for Neck Tuberculous Lymphadenitis: A Case Report

**DOI:** 10.7759/cureus.57382

**Published:** 2024-04-01

**Authors:** Hideo Ota, Hiromitsu Hoshino, Ryu Jokoji, Yoshifumi Arisaka, Hitoshi Mizuno

**Affiliations:** 1 Department of Gastroenterological Surgery, Nippon Life Hospital, Osaka, JPN; 2 Department of Gastroenterological Surgery, Itami City Hospital, Itami, JPN; 3 Department of Pathology, Nippon Life Hospital, Osaka, JPN; 4 Department of Gastroenterology, Nippon Life Hospital, Osaka, JPN

**Keywords:** rapid growth, metaplasia, tuberculous lymphadenitis, pancreas, adenosquamous carcinoma

## Abstract

Cancer (including pancreatic cancer) can develop following a *Mycobacterium tuberculosis *infection within one year of tuberculosis infection. However, it is unclear whether tuberculosis infection increases the risk of developing adenosquamous carcinoma of the pancreas (ASCP), an extremely rare cancer with a poorer prognosis than pancreatic ductal adenocarcinoma (PDAC). Herein, we report a case of rapid growing ASCP discovered upon a resection for neck tuberculous lymphadenitis. The patient was a 57-year-old woman. An excisional biopsy of the swollen right neck lymph nodes revealed tuberculous lymphadenitis. One month after the biopsy, an abdominal computed tomography scan showed a 2.0 cm (diameter) ischemic tumor in the pancreatic tail. The tissue obtained using endoscopic ultrasonography-guided fine-needle aspiration led to the pathological diagnosis of ASCP. Two months after the biopsy, the tumor had grown to 3.5 cm (diameter), and invasion of the stomach and colon was suspected. Distal pancreatectomy, splenectomy, partial gastrectomy, and transverse colectomy were performed. The final diagnosis was ASCP (4.7 cm, pT3, pN0, cM0, and pStage IIA). Postoperative adjuvant combination chemotherapy combined with antituberculosis drugs was administered orally. We report the first case of rapidly growing adenosquamous carcinoma resected from the pancreas in association with tuberculous lymphadenitis. Additional evidence is needed to confirm that tuberculosis infection increases the risk of developing pancreatic adenosquamous cell carcinoma because its potential role in promoting squamous metaplasia is unclear.

## Introduction

Cancer and tuberculosis are two of the most common diseases affecting human health worldwide. According to the World Health Organization, tuberculosis is among the most common infectious diseases worldwide, causing 1.5 million deaths in 2018 [1] and 155 million survivors in 2020 [2]. Infectious pathogens are carcinogens that cause 2.2 million cancers globally each year [3]. Recent systematic reviews have shown an association between tuberculosis and pulmonary [4] and non-pulmonary [5] cancers. However, guidelines for cancer screening in patients with tuberculosis are lacking.

Luczynski et al. [6] showed that tuberculosis was associated with an increased pooled standard incidence ratio (SIR) of pancreatic cancer (relative risk (RR): 1.58, 95% confidence interval (CI): 1.29-1.93, I2 = 0%). Additionally, in a meta-analysis, Leung et al. [5] reported that tuberculosis was associated with an increased risk of 10 cancer types, namely, head and neck, hepatobiliary, lung, gastrointestinal, pancreatic, kidney, bladder, and ovarian cancer, as well as Hodgkin's and non-Hodgkin's lymphoma and leukemia. Moreover, Luczynski et al. [6] indicated that the SIR of all cancers was highest within the first year following tuberculosis infection (SIR: 4.70, 95% CI: 1.80-12.27; I2 = 99%). Thus, monitoring carcinogenesis and cancer development, including that of pancreatic cancer, within the first year of tuberculosis infection appears to be important.

Adenosquamous carcinoma of the pancreas (ASCP) is an extremely rare subtype of pancreatic neoplasia [7] that accounts for approximately 1-4% of exocrine pancreatic malignancies [8]. Histologically, ASCP comprises at least 30% of malignant squamous cell carcinomas with coexisting ductal adenocarcinomas [9]. The prognosis of ASCP is poor, with a reported median survival of four months [9]. Moreover, no treatment guidelines have been established.

ASCP closely associated with tuberculosis has yet to be reported. Moreover, the association between tuberculosis infection and adenosquamous carcinoma development has not been determined. Thus, we present a case of rapidly growing adenosquamous carcinoma of the pancreatic tail that was discovered sequentially after cervical tuberculous lymphadenitis.

This article was previously posted on the Research Square preprint server on March 7, 2024.

## Case presentation

**Figure 1:**
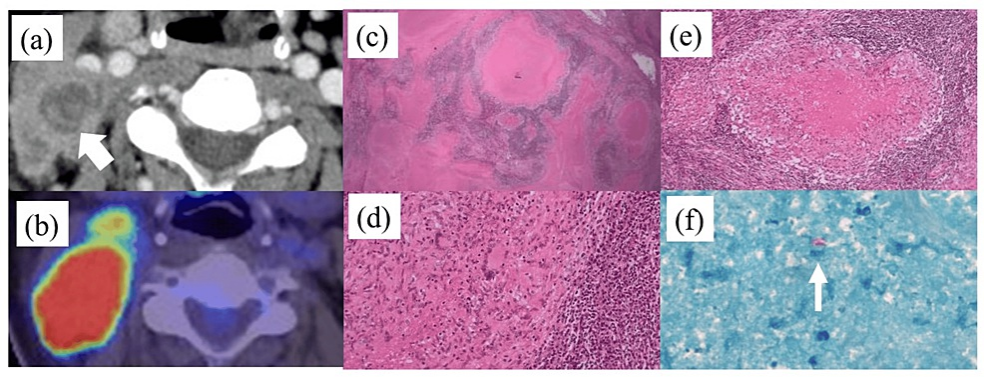
Cervical tuberculous lymphadenitis (a) Cervical enhanced computed tomography (CT) findings revealing a 10-mm (in diameter) hypovascular lesion in the swollen right cervical area (arrow). (b) 18F-fluorodeoxyglucose (FDG) positron emission tomography/CT image showing tumor uptake (SUVmax = 20.8). (c-e) Pathological examination of resected cervical lymph nodes showing epithelioid granuloma with caseation necrosis. (f) Ziehl–Neelsen staining depicting a small number of acid-fast bacterial cells (arrow), establishing a diagnosis of tuberculous lymphadenitis (50×). SUVmax: maximum standardized uptake value.

A 57-year-old woman underwent lymphadenectomy for a swollen right cervical lymph node. Pathological examination revealed an epithelioid granuloma with caseous necrosis. Ziehl-Neelsen staining revealed acid-fast bacterial cells, leading to a diagnosis of tuberculous lymphadenitis (Figure 1).

**Figure 2:**
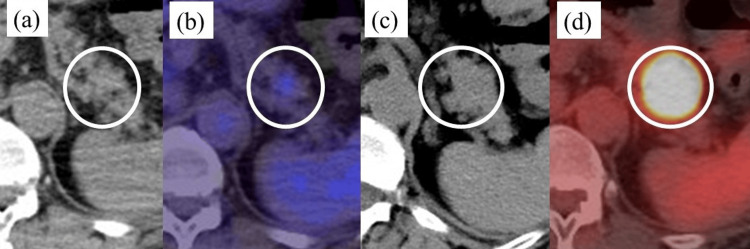
Abdominal plain computed tomography (CT) and 18F-fluorodeoxyglucose (FDG) positron emission tomography (PET)/CT (a-b) At the onset of tuberculous lymphadenitis. (c-d) Three months after the onset of lymphadenitis (during the first visit to our department). (a and c) Abdominal CT. (b and d) 18F-fluorodeoxyglucose (FDG) positron emission tomography (PET)/CT. At the onset of tuberculous lymphadenitis, 18F-FDG PET/CT confirmed a mild uptake area in the pancreatic tail (SUVmax = 2.8). Three months after the onset of lymphadenitis, this area enlarged in plain abdominal CT, and PET/CT confirmed increased uptake in this area (early SUVmax = 37.37, delayed SUVmax = 50.81). SUVmax: maximum standardized uptake value.

Three months later, abdominal enhanced computed tomography (CT) revealed a 20-mm-in-diameter hypovascular lesion adjacent to the stomach in the pancreatic tail, which was not apparent at the onset of tuberculous lymphadenitis (Figures 2A, 2C). 18F-fluorodeoxyglucose (FDG) positron emission tomography/CT confirmed significant uptake in the tumor region despite no initial uptake in this area during tuberculous lymphadenitis onset (Figures 2B, 2D).

**Figure 3:**
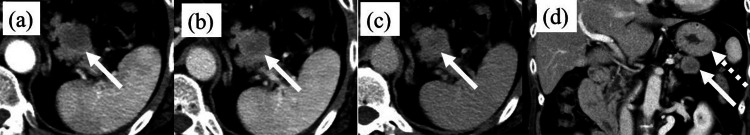
Abdominal contrast-enhanced computed tomography (CT) during the first visit to our department (a) Arterial phase; (b and d) portal phase; (c) equilibrium phase. (a-c) Abdominal enhanced CT image showing a 20-mm-in-diameter hypovascular lesion (tumor, arrow). (d) Lesions adjacent to the stomach (dotted arrow) in the pancreatic tail (tumor, arrow).

The patient was referred to our hospital for further investigation. Laboratory tests revealed no peripheral blood abnormalities. The serological results suggested latent hepatitis B infection (hepatitis B surface antigen (HBsAg) (-) < 0.001 IU/ml (0-0.004), hepatitis B surface antibody (HBsAb) (+) 341 mIU/ml (0-9), and hepatitis B core antibody (HBcAb) (+) 163.7 C.O.I. (0-0.9)) and were negative for hepatitis C and syphilis. A T-cell spot test for tuberculosis infection (T-SPOT TB) was positive. Moreover, the multiplex polymerase chain reaction was positive for *Mycobacterium tuberculosis* in tumor specimens of neck tuberculous lymphadenitis. However, the multiplex polymerase chain reaction was negative for *Mycobacterium tuberculosis*, *Mycobacterium avium*, and intracellular bacteria in three consecutive sputum samples. Based on these findings, a pre-existing tuberculosis infection was suspected. Carbohydrate antigen 19-9 (CA19-9) and squamous cell carcinoma (SCC) antigen levels were above the following normal limits: CA19-9 = 318 ng/ml (0-37) and SCC = 3.0 ng/ml (0-2.5). Carcinoembryonic antigen (CEA), duke pancreatic monoclonal antigen type 1, and serum pancreatic antigen type 1 levels were within normal limits. Abdominal enhanced CT revealed a 20-mm-in-diameter hypovascular lesion adjacent to the stomach in the pancreatic tail (Figure 3).

**Figure 4:**
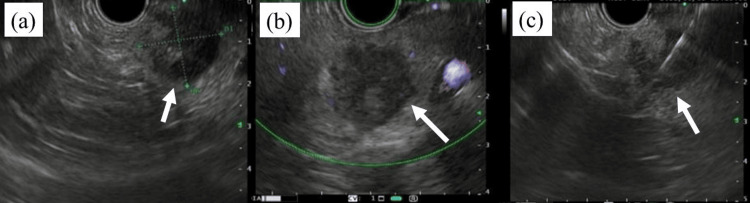
Endoscopic ultrasonography (EUS) (a) EUS image showing a 20-mm-in-diameter circular hypoechoic mass in the pancreatic tail (arrow). (b) A hypoechoic lesion is observed (arrow). (c) EUS-guided fine-needle aspiration revealing a poorly differentiated adenocarcinoma that differentiated into the squamous epithelium (arrow).

Endoscopic ultrasonography (EUS) revealed a 20-mm-in-diameter circular hypoechoic mass in the pancreatic tail. Specimens obtained from EUS-guided fine-needle aspiration (FNA) revealed a poorly differentiated adenocarcinoma with squamous metaplasia (Figure 4).

**Figure 5:**
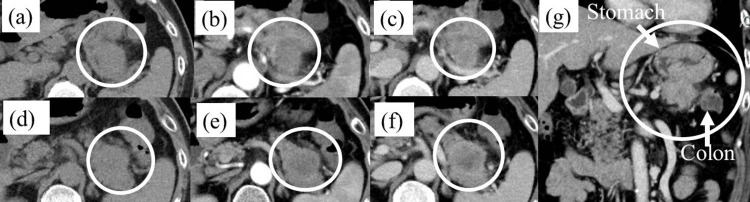
Abdominal contrast-enhanced computed tomography (CT) one month after the first visit to our department (a and d) Plain; (b and e) arterial phase; (c, f, and g) portal phase; (a-f) axial section; (g) coronal section. Abdominal enhanced CT image showing a 35-mm-in-diameter hypovascular enlarged lesion in the pancreatic tail in contact with the stomach ((a-c and g), circled area) and adjacent to the transverse colon ((d-g), circled area).

One month after the first visit, abdominal enhanced CT showed a 35-mm-in-diameter hypovascular enlarged lesion in the pancreatic tail in contact with the stomach and adjacent to the transverse colon (Figure 5).

**Figure 6:**
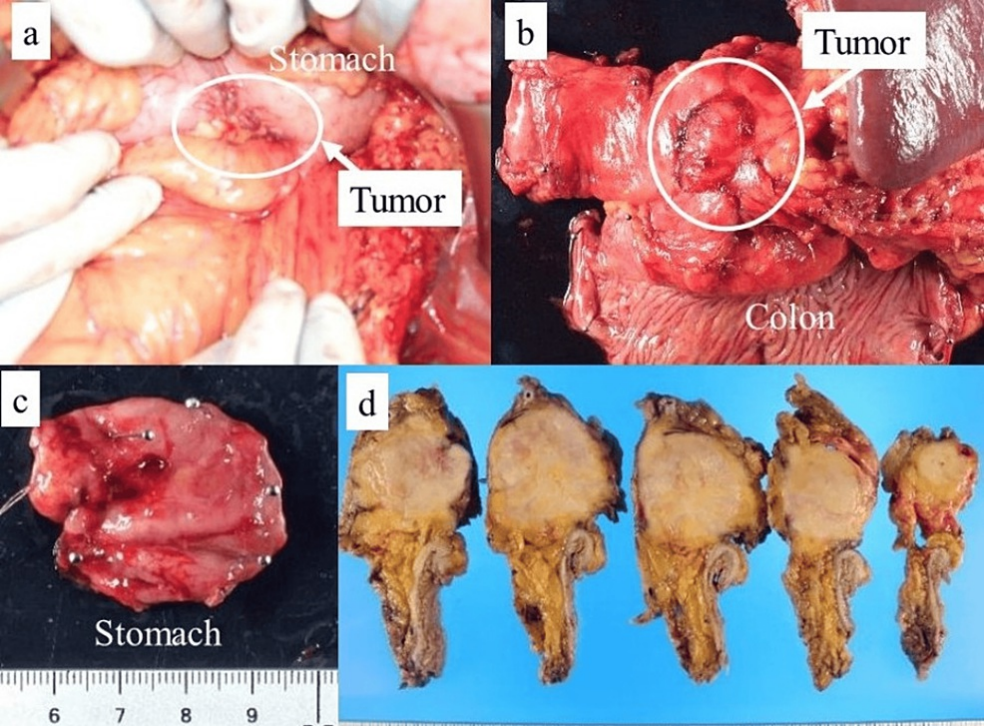
Intraoperative and macroscopic findings Distal pancreatectomy with splenectomy and partial gastric and transverse colon resection were performed. (a) Intraoperative findings indicating tumor invasion of the gastric serosa. (b) Macroscopic examination revealing tumor invasion of the gastric mucosa. (c) Macroscopically suspected tumor invasion of the transverse colon serosa. (d) Cross-section of the macroscopic findings showing tumor invasion in the transverse mesocolon without invasion of the transverse colonic serosa.

Based on these findings, adenosquamous cell carcinoma of the pancreatic tail with suspected invasion of the stomach and transverse colon was the presumed preoperative diagnosis. Therefore, we performed distal pancreatectomy and splenectomy with partial gastric and transverse colon resection (Figure 6).

**Figure 7:**
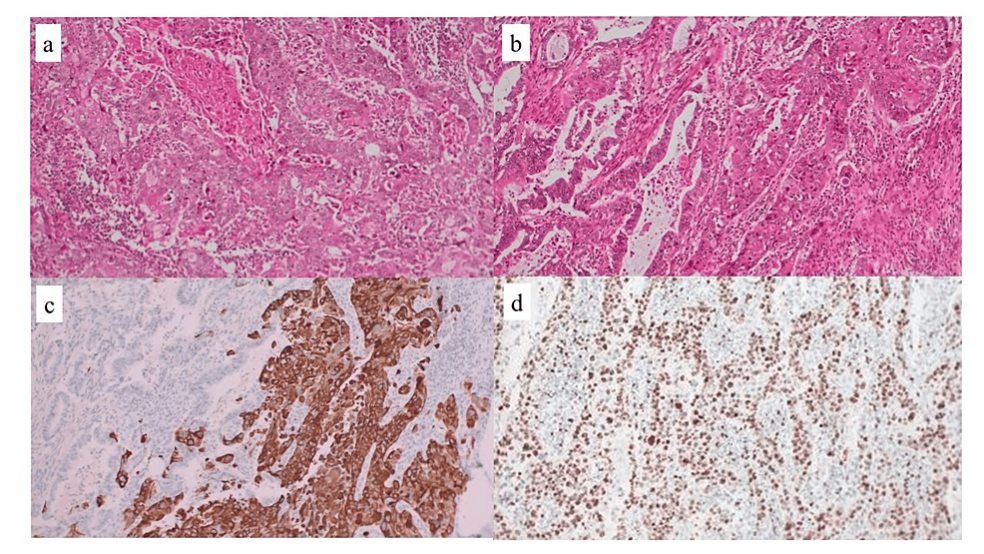
Microscopic findings (a) Atypical cells with a high degree of nuclear atypia forming alveolar structures that grow invasively, indicating squamous cell carcinoma with extensive keratinization. (b) Adenocarcinoma-forming ductal structures were observed in certain areas. The transition from adenocarcinoma to squamous cell carcinoma has been observed in some regions. Squamous cell carcinoma cells account for more than 90% of all tumor cells. (c) The transition from adenocarcinoma to squamous cell carcinoma has been observed in this region. Immunohistochemistry of CK5/6 was positive in squamous cell carcinoma and negative in adenocarcinoma. (d) Ki-67 labeling index within the viable carcinoma was approximately 80-90%.

Pathological examination revealed squamous cell carcinoma with extensive keratinization, adenocarcinoma with a ductal structure, and adenocarcinoma-to-squamous cell carcinoma. Squamous cell carcinoma cells accounted for more than 90% of all tumor cells (Figures 7A-7C). The Ki-67 labeling index within the viable carcinoma was approximately 80-90% (Figure 7D).

Based on these findings, the final diagnosis was ASCP with the following characteristics: Pt (pancreatic tail), ACS, nodular type, pTS3 (4.7 cm), pT3, int, INF-beta, ly1, v1, ne1, mpd0, pS1 (transverse mesocolon), pRP1, pPV0, pA0, pPL0, pOO1 (stomach), pPCM0, pDPM1, pN0 (0/38), cM0, and pStage IIA. The patient received a combination of an antituberculous agent with isoniazid, rifampicin, ethambutol, and pyrazinamide and adjuvant chemotherapy with tegafur/gimeracil/oteracil (Taiho Pharmaceutical Company, Tokyo, Japan). The patient was alive and had no recurrence 15 months after distal pancreatectomy and splenectomy.

## Discussion

Individuals with tuberculosis have an increased risk of both pulmonary and nonpulmonary cancer [6].

Although the pathogenesis underlying the increased risk of malignancy following *Mycobacterium tuberculosis* infection remains unknown, several hypotheses have been proposed. Tuberculosis may promote oncogenesis through chronic inflammation, particularly by increasing circulating tumor necrosis factor-alpha (TNF-ɑ) levels. This factor improves tumor cell survival via antiapoptotic intracellular signaling pathways, angiogenesis, and mutagenesis [10]. Tuberculosis leads to fibrous scar formation in the lungs and is associated with an increased incidence of cancer over time [11]. This is partly due to impaired lymphatic flow, which causes decreased immune surveillance and increased deposition of metastatic cells [12]. Grivennikov and Greten review the mechanisms underlying the initiation of pro-tumorigenic inflammatory responses, how these evolve throughout the different stages of tumor development, and the plasticity of the cells within the tumor microenvironment [13]. These hypotheses are likely to apply not only to lung cancer but also to other types of cancer.

Leung et al. [5] and Luczynski et al. [6] reported that tuberculosis is associated with an increased risk of pancreatic cancer. However, the factors contributing to the development of adenosquamous carcinoma after *Mycobacterium tuberculosis* infection have not been identified.

Nalbandian et al. [14] showed that squamous cell aggregates consistently appeared within the lung tissue associated with chronic tuberculous lesions and, in some cases, resembled SCC. Chronic tuberculous infection induced lung-specific cell dysplasia and resulted in SCC formation in an experimental model. Tuberculosis may result in the reactivation of permanent tuberculous infection, severe tissue damage, and the appearance of squamous metaplasia in the lung and several other tissues. However, further evidence is needed to clinically confirm the findings from this experimental model.

Hypotheses regarding the developmental mechanism of adenosquamous cell carcinoma include its origination from cells capable of differentiating into columnar and squamous epithelium [15], the possibility of ectopic squamous epithelium or normal pancreatic duct metaplasia becoming cancerous [16], and the transformation of adenocarcinoma into squamous cell carcinoma (squamous metaplasia of adenocarcinoma) [17]. These three hypotheses support the squamous metaplasia theory of adenocarcinomas, indicating that pancreatic adenocarcinoma develops first, followed by squamous cell metaplasia of adenocarcinoma cells. Pathology revealed a transitional area between the adenocarcinoma cells and the squamous epithelium, which could indicate squamous metaplasia of the adenocarcinoma.

Previous studies indicate that tuberculosis infection increases the risk of developing pancreatic adenocarcinoma, while adenocarcinoma cells can undergo squamous metaplasia favored by factors such as infection and the existence of adenosquamous cell carcinoma per se. However, this might be just a coincidence, and not be related to the development of pancreas carcinoma. This evidence is insufficient, and further studies are required to determine whether tuberculosis infection affects the progression of pancreatic adenocarcinoma to squamous metaplasia.

Charbit et al. demonstrated that the doubling time of squamous cell carcinoma patients is approximately 80 days, which is half that of adenocarcinoma patients [18]. Borazanci et al. reported that tumor cell necrosis is frequently observed in patients with adenosquamous carcinomas [19]. Necrosis of tumor cells may lead to expansive growth. The area of squamous metaplasia probably increased rapidly due to expansive growth.

According to the matched pair analysis for treatment and prognosis of ASCP compared to pancreatic ductal adenocarcinoma (PDAC) by Kaiser et al. [20], the five-year overall survival (OS) rates were comparable, with 18.2% in the ASCP group (n = 91) and 17.5% in the PDAC group (n = 2653); however, the median OS was significantly poorer in patients with ASCP than in the unmatched cohort of patients with PDAC. Moreover, the OS of all patients who underwent resection for ASCP (n = 83) was significantly longer than that of the patients who did not undergo resection (n = 6). Furthermore, the median survival of patients who received adjuvant chemotherapy (n = 52) was significantly longer than that of patients who did not receive adjuvant chemotherapy (n = 24). Patients who do not experience early recurrence after resection or adjuvant chemotherapy for the treatment of ASCP may have a five-year survival rate similar to that of patients with PDAC.

## Conclusions

We report the first case of a rapidly growing adenosquamous carcinoma that developed in the pancreatic tail sequentially with the development of cervical tuberculous lymphadenitis. Patients with tuberculosis are more likely to develop cancer within one year of infection diagnosis. Although tuberculosis may induce the development of pancreatic adenosquamous cell carcinoma, its effect on squamous metaplasia has been demonstrated only at an experimental level and has not been fully elucidated; thus, further evidence is needed.
